# Supplementary material for: Are carbon emissions trading and green financial instruments synergistic? -Comprehensive quantitative research based on content analysis
Source: PLoS One. 2024 Mar 7;19(3):e0298601. doi: 10.1371/journal.pone.0298601 (PMC10919601; doi:10.1371/journal.pone.0298601)
Supplement: S1 Data — (ZIP) [file pone.0298601.s001.zip › Data/地级市Z_F工作报告.zip!/╔╜╢1⁄2/╟α╡║╩╨/└┤╫╘╣1⁄2╓┌║┼í╛┬φ┐╦╩2╛▌═°í┐_2005.docx]

各位代表：

　　现在，我代表市人民政府向大会作工作报告，请予审议，并请各位政协委员和其他列席的同志提出意见。

　　一、2004年工作回顾

　　过去的一年，是我市改革开放和现代化建设取得显著成效的一年。在中共青岛市委的正确领导下，我们紧紧依靠全市人民，以邓小平理论和“三个代表”重要思想为指导，贯彻落实党的十六大和十六届三中、四中全会精神，贯彻落实省委、省政府的各项部署，以科学发展观统领全局，按照市委“五个三”的工作思路，全力推进各方面工作，较好地完成了市十三届人大二次会议提出的各项任务，“十五”计划确定的主要目标提前一年基本实现。

　　初步核算，全市生产总值完成2163.8亿元，增长16.8％；地方财政一般预算收入130.5亿元，增长28.5％。全社会固定资产投资1025.4亿元，增长38.7％。三次产业的比重为7.5：54.1：38.4。

　　（一）认真落实宏观调控政策，经济结构调整步伐加快

　　宏观调控取得阶段性成效。全面清理整顿各类开发区，规范土地市场秩序，全市累计核减开发区规划用地面积67.2％，整合挖潜存量土地4.8万亩，争取国家确认重点急需建设项目用地2.9万亩。采取积极措施，克服了煤电油运方面的困难。全年实现规模以上工业增加值增长26.9％。高新技术产业产值增长35％，占规模以上工业总产值比重超过40％。

　　加快构建可持续发展的经济体系，着力培育六大产业集群，大项目和品牌经济取得新进展。1000万吨炼油项目获国家批复即将开工。与中船重工集团签署全面合作协议。前湾港三期、海西湾造修船基地和青岛发电厂二期扩建工程等项目进展顺利。丽东化工、LG手机等项目开工建设。高丽钢线三期、浦项不锈钢等项目建成投产。我市被确定为首批国家电子信息产业基地。海尔手机项目竣工达产。16个产品获得“中国名牌产品”称号。新增“中国驰名商标”4件。

　　现代服务业繁荣发展。马士基中国北方总部、大韩航空中国呼叫中心分别落户我市。港口吞吐量达到1.6亿吨，集装箱吞吐量突破500万标箱。航空旅客吞吐量480.8万人次，航空货邮吞吐量10.5万吨。极地海洋世界、现代艺术中心、香格里拉大饭店二期奠基开工。旅游总收入、海外游客、国内游客的人数分别突破200亿元、50万人次和2000万人次。引进恒丰银行、大众保险等5家金融机构，金融业实现地方税收增长21.4％。商贸业对半岛地区辐射能力进一步增强。

　　（二）实施城乡统筹发展，努力增加农民收入

　　市本级安排6.84亿元用于“三农”，增长28.6％。各区全部免征农业税，郊区五市降低农业税税率3个百分点，减免农业税及附加2.3亿元，向农民发放粮食直补资金4424万元。全年粮食总产232.3万吨，增长4.6％。农民人均纯收入5080元，增长12.1％，增幅达1998年以来的最高水平。历年拖欠农民的土地安置补偿资金，全部补偿到位。农业产业化加快发展，新增国家级龙头企业3家，累计达到9家。郊区五市全部进入2004年全国100强县。高致病性禽流感防治工作扎实有效，保证了全市安全无疫情。

　　（三）加快推进城市建设，城市环境进一步改善

　　城市规划和重点建设取得新进展。城市总体规划修编全面展开。编制完成滨海公路沿线城市组团总体规划、生态市建设规划、城市综合交通规划等专业规划，7区控规覆盖率达到100％，5个区的分区规划开始实施。前湾港区港口总体规划修编已通过国家主管部门审查。“三点布局、一线展开、组团发展”的城市框架全面展开。滨海公路北段、滨海步行道崂山区段、石老人海水浴场改造开工建设。胶济线青岛段铁路电气化改造加快实施。青银高速公路二期工程奠基。汇泉广场改造、流亭机场扩建工程竣工并投入使用。“平改坡”试点工程基本完成。跨海隧道项目完成地质勘探、专家论证和社会公示、听证，南隧、北桥建设方案进入报批程序。

　　《奥运行动规划》顺利实施。奥帆赛场馆建设及相关配套工程全面启动。观摩了雅典奥运会和国内外大型帆船比赛活动的组织管理工作。在国内外开展了“帆船之都”推介活动。

　　生态市建设开始启动。顺利通过国家环保模范城市省级复查。工业废水排放达标率、烟尘控制区覆盖率居全国领先水平，城市环境空气质量优良率达90.7％。前海一线截污工程加快实施。全面开展新一轮城市环境综合集中整治。完成市内四区12条主要道路绿化和237条道路的路灯安装工程，新植树69万株。建成区绿化覆盖率38％。全年清除违法建筑114.3万平方米。实施清洁生产，推广资源综合利用，循环经济发展取得局部成效。

　　（四）不断深化各项改革，对外开放迈出新步伐加快推进各项改革。年内完成了市属国有和集体中小企业整体出售和股权转让55户。132户国有中小企业完成改制退出程序，改退率75.5％。搭建国企改革资金平台，筹集资金14.9亿元，安置职工8.1万人。着手开展国有（集体）资产监管工作，对27户市直属企业实施监管。农村信用社改革取得新的进展。市与区市财源建设联动机制基本建立。积极稳妥操作，保证了出口退税改革的顺利实施。深化政府机构改革，初步理顺了行政管理体制。

　　民营经济实力明显增强。实现增加值、税收分别增长18％和22％，出口增长93.8％。年销售收入过1亿元、10亿元的企业分别达到148户和13户。12户企业进入全省民营100强企业。民营企业上市融资实现新突破，中天国际在香港联交所挂牌，高校软控已获准上市。

　　对外开放水平进一步提高。完成外贸进出口总额243.3亿美元，其中出口139.1亿美元，增长37.3％，实际利用外资38.3亿美元，增长33.1％。创新招商方式，重点推进产业集群招商和专业招商。全年引进千万美元以上大项目212个，世界500强企业新投资项目11个。成功举办“韩国周”、“日本周”和“瑞典日”活动。实际利用韩、日投资分别增长46.6％和10.2％。积极转变外贸增长方式，高新技术产品和机电产品出口分别增长53.6％和50.3％。实现对外经济合作营业额2.1亿美元。“区港联动”试点获国务院批准。出口加工区正式运行。外事工作服务国家外交大局，对外务实交往取得新成绩，与国外城市的交流与合作进一步扩大。港澳台侨工作得到加强。成功承办了亚洲合作对话第三次外长会议、第三届APEC中小企业技术交流暨展览会和全国国有企业领导班子思想政治工作建设座谈会等一系列重要会议。引进国内投资192.7亿元。中船重工、中石化等一批国家重点企业和北京首创、上海申沃、国美、苏宁等知名企业落户青岛。加强了对省内外对口帮扶地区的支援。

　　（五）深入开展城乡文明建设，社会各项事业协调进步

　　创建全国文明城市工作富有成效。广泛开展“双学三创”活动。对市群众艺术馆和市图书馆进行了改造，少儿图书馆正式对外开放。我市再次荣获全国无偿献血先进城市奖。深入开展“双拥”共建活动，地方政府积极实施拥军“四项工程”，有力地支持了部队国防建设。驻军广大指战员全力参与抢险救灾、扶贫帮困，协助地方发展公益事业和承办重大活动，为青岛的发展与稳定作出了重要贡献。国防教育、民兵预备役建设不断加强，人防工作坚持平战结合取得新进展。中央、省驻青单位热情关注和大力支持青岛的建设与发展，做了大量积极有效的工作。

　　社会事业协调发展。8项成果获国家级科学技术奖。专利授权量1973件，增长32％。我市被列为全国企业技术创新试点城市。国家深海潜水器基地定点我市。全面实施了义务教育“一费制”，“以县为主”的农村义务教育管理体制进一步完善。全市初中毕业生升学率达到92％。职业教育继续得到加强。取消了市内四区外来务工人员子女借读费。基础教育新课程改革试点全面启动。市立医院东院区主体完工。青岛大剧院完成方案设计。市体育馆已开工建设。北京电影学院青岛教学基地奠基。全市有313项文化创作精品在全国获奖。实现有线数字电视整体转换15万户。我市运动员在第28届雅典奥运会和第12届残疾人奥运会上取得历史最好成绩。人口低生育水平继续保持稳定。妇女儿童、老龄工作稳步推进，残疾人和红十字会事业健康发展。气象、地震、民族、宗教、档案、史志等工作取得新成绩。

　　（六）着力解决事关群众切身利益的实际问题，人民生活水平稳步提高

　　市政府年初确定重点办好的17件实事，除香港中路人行过街通道等个别项目调整外，其他都顺利完成或达到进度要求。继续保持就业增速高于失业增速的良好势头，全年实现就业14.1万人，城镇登记失业率3.1％。城市居民人均可支配收入11089元，增长10.1％。新增农村劳动力转移就业15.7万人。拖欠农民工工资问题得到基本解决。降低了国有集体企业养老保险缴费比例，参保缴费职工突破100万人。农村新型养老保险制度试点进一步扩大。提高了城乡最低生活保障标准。全面推行新型农村合作医疗制度，农民受益面达到89％。经济适用房竣工64.8万平方米。市区新增供热面积157万平方米，新增天然气用户8.5万户。城区北部开工建设了辽阳西路、安顺路、李村河等一批道路、河道整治项目。对“市民月”活动中集中提出的缩小南北差距、加强城市管理等10大问题，制定了三年工作计划，正在逐步推进。加强对粮食、蔬菜等商品价格的监控，增加重要商品储备，市场物价保持平稳。

　　（七）大力推进“平安青岛”建设，保持了社会政治稳定

　　以创建“平安青岛”为目标，坚持打防并举、预防为主，完善了打、防、控一体的社会治安管理机制。坚持重心下移，抓基层、抓基础、抓队伍，健全了基层创建平安工作机制。社会治安综合治理责任制得到有效落实，进一步强化了维护社会稳定的领导机制和责任体系，创建“平安青岛”工作取得阶段性成果。刑事案件立案、治安案件受理分别下降5.6％、32.1％。及时侦破了几起重大刑事案件，全市公众的治安满意率进一步提高。

　　在全国率先开通了公共信息网络安全报警处置中心。加强公共安全应急体系建设，建立完善了38个专项应急预案。全市各类安全事故起数、死亡人数分别下降32.5％、21.3％。实施“食品药品放心工程”，全市食品药品安全形势明显好转。重视群众来信来访和矛盾纠纷排查调处工作，及时化解不安定因素，促进了社会的和谐稳定。

　　（八）全面推进依法行政，政府自身建设得到加强

　　广泛开展《宪法》、《行政许可法》和《全面推进依法行政实施纲要》的学习、宣传、贯彻活动。对地方性法规和政府规章进行全面清理，报请市人大常委会废止8件、修改19件地方性法规，废止114件、修改37件政府规章。完成了行政许可事项和实施主体的清理工作。在城市管理中全面推行了相对集中行政处罚权。深入开展“三快一提高”和“三访谈”活动，严肃查处公务员不作为、乱作为的行为，促进了政府工作作风的转变。自觉接受市人大、政协和人民群众的监督，人大代表建议和政协委员提案全部办理完毕，第6号、第10号议案完成阶段性落实工作。

　　各位代表！过去一年的成绩来之不易。这是认真贯彻落实中央和省委、省政府各项工作部署的结果，是市委正确领导、市人大和市政协支持监督的结果，是全市人民团结奋斗、共同努力的结果。在此，我代表市人民政府，向全市广大干部群众，向各民主党派、工商联、人民团体和社会各界人士，向中央、省驻青单位，向人民解放军和武警驻青部队，向所有关心、支持青岛建设和发展的海内外朋友们，表示衷心的感谢和崇高的敬意！

　　各位代表！我们也清醒地看到，当前我市经济社会发展中还存在许多矛盾和问题，经济结构调整和产业升级任务依然艰巨，服务业发展相对滞后，民营经济比重偏低；某些方面的体制、机制性障碍还很突出，经济增长方式比较粗放，生态环境压力较大；城市建设和管理体制机制需要理顺，城乡差距和南北差距依然较大；优质教育和卫生资源供求矛盾突出，文化体育设施薄弱；城乡居民整体收入水平不高，农民增收的长效机制尚未建立，就业和社会保障面临较大压力；政府作风建设还存在薄弱环节，依法行政意识需要增强，执行能力和行政效能有待提高，不正之风和腐败现象仍有发生。对于上述问题，我们将高度重视，通过发展和改革逐步加以解决。真诚希望各位代表、政协委员和社会各界、广大人民群众给予监督，帮助我们把政府工作做得更好。

　　二、2005年工作的主要任务

　　2005年是贯彻落实科学发展观，巩固宏观调控成果，全面完成“十五”计划目标的重要一年。今年政府工作的总体要求是：以邓小平理论和“三个代表”重要思想为指导，认真贯彻党的十六大和十六届三中、四中全会精神，全面落实科学发展观，坚持发展这个第一要务，围绕建设全国重点中心城市和世界知名特色城市的目标，着力推进改革开放，加快调整经济结构，转变经济增长方式，突出抓好“三件大事”，大力推进“繁荣青岛、平安青岛、文明青岛”建设，努力构建社会主义和谐社会，实现经济社会全面协调可持续发展。

　　经济和社会发展的主要预期指标是：全市生产总值增长14％，地方财政一般预算收入增长14％；城市居民人均可支配收入、农民人均纯收入分别增长10％和7％；城镇登记失业率控制在4.2％以内，居民消费价格总指数调控在104以内。

　　各位代表！做好今年的各项工作，我们既面临着多方面的挑战，也面临着难得的发展机遇。国家将继续实行宏观调控政策，实施稳健的财政和货币政策；省委、省政府突出青岛龙头带动作用，胶东半岛制造业基地和山东半岛城市群建设步伐进一步加快；市委“五个三”的工作思路全面展开，科学发展、快速发展、和谐发展的态势已经形成。只要我们切实增强机遇意识、危机意识、创新意识，紧紧依靠全市人民，万众一心，努力拼搏，就一定能够完成各项目标任务。

　　（一）继续推进经济结构调整，坚持走新型工业化道路

　　加快先进制造业基地建设。围绕构建可持续发展的经济体系，着力培育六大产业集群，推进炼油、造船、汽车等20个投资10亿元以上大项目、100个投资过亿元的重点项目。电子和家电产业，要加快国家电子信息产业基地和园区建设；石化产业，要加快1000万吨炼油、丽东化工等主体项目建设；造船产业，要加快海西湾造修船基地建设进度，做好船舶研发、装备设计与制造等项目引进；汽车产业，要抓好货车、乘用车、特种车的生产，加快重要零部件和研发中心的建设；全面整合港口资源，加快前湾港区的建设，规划新建鳌山港。

　　加强大企业和品牌梯队建设。制定扶持大企业本地发展政策，完善直通车服务。积极扶持有条件的大企业向世界500强迈进，推动一批重点企业进入国内同行业前列，培植一批民营企业成为全国民企百强。引导中小企业围绕产业集群建设，形成配套加工体系。大力发展品牌经济，支持一批销售收入过10亿元的企业实施品牌培育。加强工业经济运行的监控和指导，协调好煤电油运等生产要素供应。

　　积极推进现代服务业发展。壮大港口经济，推进“区港联动”，提高口岸服务水平，提升国际货物中转和分拨功能，促进港航产业与物流业互动发展。以建设国际海滨度假城市为目标，全面推进旅游业向度假型、质量型和国际标准的转变。开发整合旅游资源，培育和完善度假旅游、节会旅游、生态旅游等系列旅游产品，加强极地海洋世界等重点旅游项目建设，进一步加大国内外促销力度。加快会展中心二期工程建设，高质量办好青岛国际啤酒节、青岛海洋节、中国国际消费电子博览会等重要节会。争取有两家国内外金融机构落户。大力发展总部经济，吸引更多的大公司地区总部进驻我市。完善市、区（市）两级商业网点规划管理体系，加快培育社区商业中心和区域性商贸中心。

　　（二）保持农业和农村发展的良好势头，继续增加农民收入

　　推进城乡统筹发展，围绕发展郊区特色经济，切实加强农业综合生产能力建设，全面深化农村改革，进一步促进粮食稳产、农民增收、农村经济社会协调发展。

　　加快农业结构调整。抓好100万亩高产优质粮食示范区建设。大力发展畜牧、水产、蔬菜、果品、花卉等优势产业。实施农业科技创新，提高农业科技含量。抓好高效农业园区、农产品出口基地和无公害农产品生产基地建设。全市粮食总产230万吨以上。推进十大农业园区和十大农产品专业批发市场建设，重点扶持9户国家级农产品龙头企业。大力推广“九联模式”。支持发展各类农民专业合作经济组织，提高产业化经营水平和农民组织化程度。建成青岛国际农产品展示交易中心。实施“绿卡行动计划”，加强质量检测防疫体系建设和优质无公害农产品认证管理。做好重大动植物疫病的防控工作。

　　进一步加强农村基础设施建设，提高农村城市化水平。增加对大沽河治理、节水示范、灌区配套等项目的资金支持。实施墨水河综合治理一期工程。拓展农业项目投融资渠道。认真做好全国重点镇、卫星镇、小康村的示范工作，组织好国家级小城镇发展改革试点和小城镇经济综合开发工作。

　　认真落实党的农村基本政策。进一步减轻农民负担，努力增加农民收入。取消农业税征收和农村“两工”。继续实行对种粮农民的直接补贴和农机具购置补贴。全年市本级财政用于“三农”的支出9.5亿元。深入贯彻《农村土地承包法》，稳定和完善土地承包关系。完善征地补偿办法，落实补偿监管机制，妥善安置被征地农民。落实涉农价格和收费公示制度，切实保障和维护农民的合法权益。继续加大对经济困难镇村的财政转移支付力度，专款专用，确保工资发放和农村基层组织正常运转。

　　（三）加快改革开放步伐，增强经济发展的活力和动力

　　深化国有企业和国有资产管理体制改革。重点推进国有大企业股份制改革，基本完成国有资本从中小企业改制退出和困难企业退出市场。资产经营公司基本完成过渡性体制改造。完善对大企业的监管工作，逐步将市政、公用、交通等行业符合条件的国有资产纳入监管范围。探讨建立国有资产经营预算制度，规范企业审计评估和产权交易，完善企业经营者业绩考核和激励机制，依法履行出资人职责，促进国有资产保值增值。

　　深化财税和投资体制改革。完善公共财政体制，支持农村税费改革等工作。进一步拓展财源建设途径，建立财政收入持续快速增长机制，提高财政收入占全市生产总值、税收占财政收入的比重。加大财政对“三农”工作和社会保障的支持力度。改革企业投资项目审批办法，规范政府投资行为，切实加强财政专项资金使用管理，加强投资项目的审计监督。继续做好企业上市工作。

　　大力发展民营经济。出台促进民营经济发展的政策措施，扶持一批民营科技型、外向型、现代服务业和农产品加工型企业做强做大。支持民营中小企业做专做精，为产业集群发展协作配套。加快民营企业争创品牌步伐。鼓励民营企业参与国有企业重组改造和上市融资，参与基础设施和公用事业建设。建立完善融资担保体系、服务体系，创新适应民营企业特点的金融产品和服务方式。建立促进民营经济发展协调机制，解决民营企业发展中遇到的困难和问题。全年实现民营经济增加值和税收分别增长18％、16％以上。

　　转变对外贸易增长方式。提高电子信息产品和软件等高附加值产品出口比重，引导出口产品由数量型向质量型、环保型转变，推动加工贸易转型升级。加快出口加工区发展。做好出口退税改革的相关工作，完善扶持出口的政策措施和协作机制。推进外贸企业收购制向代理制改革。组织好急需或短缺的能源、原材料、先进技术和关键设备进口。做好纺织品出口配额取消后的应对工作，提高贸易风险防范能力和贸易摩擦处理能力。引导企业加强职业安全卫生管理体系（ISO18000）认证工作。

　　进一步提高利用外资质量。强化大企业、产业集群和产业链配套项目招商，整合招商队伍，推进专业招商，做好委托招商。紧紧围绕我市产业发展方向，加强对韩国、日本、欧美等国家和港台地区引资工作。办好各项对外招商和经贸交流活动。推进服务业和市政公用设施领域的对外开放。积极开拓拉美市场，争取有所突破。进一步提高开发区发展水平，加快发展现代制造业，逐步向多功能综合型产业区转变。落实好赋予开发区的各项权限。加大国内招商力度，吸引国家重点企业和国内500强企业在青投资、设立研发基地、地区总部和销售中心。进一步实施“走出去”战略。加强政策和市场研究，优化商务环境，推广“绿色通道”等特色服务。改善外来投资创业人员的工作和生活条件。加强与国外友好城市和机构的交流与合作。做好结对帮扶曹县和支援贫困地区工作。

　　（四）加快城市建设和奥帆赛筹备，提高城市现代化水平

　　高标准做好城市规划工作。围绕加快推进现代化国际大城市框架和可持续发展的经济体系建设，搞好滨海公路畅通和两侧延伸道路规划，完成五个组团范围内的控规及《生态间隔区详细规划》，加快市中心区整治与完善规划、港口总体规划、“帆船之都”发展概念规划的编制工作。在加快城市西部发展的同时，积极开展对东部和北部地区的规划研究。

　　加强基础设施建设。通过规划指导、政策扶持，推进市区北部基础设施建设，改善生活环境，逐步缩小南北差距。实施杭州路、大沙路、开封路、四流北路、四流中路等超期服役道路的综合整治。做好鞍山路高架等海底隧道市区接线项目的前期工作。全线改造山东路，完成辽阳路综合整治工程。加快滨海公路、青银高速公路二期、济青高速南线、流亭机场二期扩建、青岛电厂扩建、仙家寨水厂老厂改造和麦岛污水处理厂扩建等重点项目建设。完成胶济线青岛段铁路电气化改造。完成本地网电话号码升8位工程。

　　理顺城市管理体制。将市政道路、园林绿化、环境卫生、房产管理等方面的主要管理权限下放到区，实行属地化管理。强化城市管理综合行政执法，建立城管执法公安保障机制。完善以市场为基础的公用事业价格形成机制和调节机制。按规定程序做好《青岛市城市房屋拆迁管理条例》的修订工作。继续清除违法建筑，建立防治违法建筑行为的长效机制。集中整顿户外广告设施、各类亭体和占路经营，深入开展创建城管示范达标路活动。

　　全面做好奥帆赛筹备工作。坚持节俭办奥运原则，完成奥帆赛必备项目主体工程，加快相关市政管线敷设和配套设施建设。实施科技奥运工程，开展科技奥运研究项目。实施“数字奥运”行动计划，奥运海底光缆项目竣工。完成赤潮防治和水文、气象监测以及国家级监测的阶段性目标。全面普及奥运及帆船运动知识，加强奥运城市宣传推介，全力打造“帆船之都”城市品牌。

　　（五）做好创建全国文明城市工作，促进社会全面进步

　　争创首批全国文明城市。对照《全国文明城市测评体系》，全面排查、整治薄弱环节。大力宣传《公民道德建设实施纲要》和“八不”行为规范，加强未成年人保护工作和思想道德教育。深化文明社区、文明行业、文明村镇等群众性精神文明创建活动。在全市服务行业广泛开展“创服务名牌，树青岛形象”活动。深入开展城市文明交通“三让”活动，树立一批示范街道、示范社区。继续推进学习型城市建设，形成广覆盖、开放式、多渠道终身教育体系。加强各级社区教育阵地建设，开展好各种形式的社区读书活动。

　　全面推进社会事业发展。启动“文化建设年”活动，实施“文化家园”工程，加快青岛大剧院、现代艺术中心等文化设施建设，抓好社区、村镇文化中心和文化大院建设，丰富群众文化生活。加强对历史文化名城和民族民间文化的保护。办好中国国际小提琴比赛和奥林匹克文化节。推进文化体制改革，加强文化市场管理，大力发展文化产业。广泛开展全民健身活动，增强竞技体育实力，高水平办好青岛市第一届运动会、中日韩国际马拉松赛等体育赛事。加快市体育馆、市体校的建设。积极推进公共卫生长效机制建设，健全和完善疾病控制、卫生监督、医疗救治三级网络体系。深入扎实开展传染病防治工作。市立医院东院区竣工交付使用。重视和支持红十字会工作。加快县级卫生城市建设步伐，调整优化市属医疗机构资源。进一步繁荣发展哲学社会科学事业，普及科学知识，倡导文明健康的生活方式。深入开展双拥共建活动，加强人防、民兵预备役工作，巩固军政军民团结。积极支持部队国防建设，解决好驻军工作生活等问题。认真实施妇女儿童发展纲要，推动妇女儿童事业不断发展。进一步做好民族、宗教、侨务、对台、老龄、档案、史志、残疾人事业等工作。

　　（六）坚持科教兴市和人才强市战略，实现可持续发展

　　提高科技自主创新能力，完善城市创新体系。加快共建国家重点实验室培育基地，开工建设国家深海潜水器基地，促进国家海洋科学技术研究中心建设。抓好国家企业技术创新试点，支持海尔、海信等企业建设国际一流的科研中心，深化制造业信息化示范工程。搞好市南、高新区、海尔、海信软件基地建设；推进新材料、新兴海洋科技产业和生物工程产业快速发展。高新技术产业产值增长20％以上。加快创新性科研成果向产业化转化，扶持和促进中小型企业开展技术创新。

　　推动城乡教育协调发展。落实农村“以县为主”的义务教育管理体制，完成农村中小学标准化建设任务。逐步将五市三区义务教育阶段教师工资、公用经费、校舍危房改造纳入区市财政预算。设立农村困难家庭高中生救助资金，完善救助制度。加强教师队伍建设，全面推进素质教育。扩大优质高中资源，大力发展各类职业教育，优化职业教育资源配置。实施农村中小学现代远程教育工程。支持青岛高校发展，加大重点高校引进力度。深入实施人才强市战略，加强高层次人才队伍建设，实施科技将才建设工程，健全高技能人才培养机制。完善人才落户政策。

　　切实做好人口、资源和环境工作。深化人口与计划生育综合改革，提高出生人口素质，人口出生率控制在11.6‰以内。全面推行农村部分计划生育家庭奖励扶助制度。认真落实国务院《关于深化改革严格土地管理的决定》，做好土地管理体制改革和土地利用总体规划修编工作。加强对耕地特别是基本农田的保护，做好建设用地的节约挖潜，制定土地集约利用考核指标体系。全面推进生态市建设。大力发展循环经济，做好省级以上工业园区循环经济发展规划编制工作；加强资源综合利用，提高清洁、可再生能源使用率，建设资源节约型社会。逐步实施现有建筑的节能改造。对青银高速、海尔路、香港路等重点道路沿线山体进行恢复和保护。抓好海洋环境保护、胶州湾湿地保护、前海重点水域、重点流域和区域污染防治。完成官路水库、沐官岛水库前期工作，加强饮用水源地保护，推进中水综合利用和海水淡化工程。重视治理空气污染，加大燃煤和机动车污染控制力度，机动车尾气排放达标率90％以上。

　　（七）坚持以人为本，做好关系人民群众利益的各项工作

　　努力扩大就业规模。全面落实促进就业各项政策，逐步建立城乡统筹就业良性互动机制。建立公益性岗位储备制度，公益性岗位总量达到1.2万个，安置就业困难人员保持在1万人。推进实施创业工程，扶持自谋职业和自主创业1万人。加强职业技能培训工作，全年培训失业人员3万人，组织农村富余劳动力转移培训3万人，培训企业技术工人3万人。从今年起，建立机电、焊接、化工环保等6个公共实训基地。提高最低工资标准，努力建立与经济增长相适应的合理增资机制。强化工资指导线作用，督促企业按时足额发放职工工资。加强劳动执法监察，维护劳动者合法权益。落实库区移民扶助政策。

　　健全社会保障体系。将国有、集体企业养老保险缴费比例降至21％。增加企业养老保险参保人数7万人以上。确保企业离退休人员养老金按时足额发放。扩大城镇职工基本医疗保险市级统筹范围，提高基本医疗保障水平，降低个人负担。全面推行农村社会养老保险制度，着力解决好失地农民的社会保障问题。建立健全城乡社会救助体系，巩固和完善新型农村合作医疗和医疗救助制度。实施农村低保“阳光救助工程”。建立城乡自然灾害援助制度。改革现行五保供养体制，加快农村中心敬老院、老年福利中心等设施建设。

　　改善城乡居民生活条件和环境。全面落实《城镇最低收入家庭廉租住房管理办法》，努力增加廉租住房供给量。加快经济适用房建设，规范销售行为，严格执行购买经济适用房的申请、审批和公示制度。实施农村贫困残疾人安居工程，年内改造1000户低保残疾人住房。完成6万户居民自来水一户一表改造。新增供热面积160万平方米，其中老城区50万平方米。完成“平改坡”一期工程。实施海岸路河、郑州路河、大村河等河流的重点治理，对“小街小巷”逐步实施道路硬化、路灯安装、雨污管道敷设。进一步做好中山路、火车站周边等地区的保护与改造。在市区主要干道两侧和山头、公园广种乔木和彩叶树种，新建城市公共绿地95万平方米，市郊完成新造林19.4万亩。基本完成市区有线数字电视整体转换。完成1000个村庄通自来水工程。加强市场物价监管，把握好公共服务产品价格调整的时机和力度，加强国计民生重要物品储备，保持市场物价的基本稳定。

　　（八）深入推进“平安青岛”建设，切实维护社会稳定

　　全力打造“平安青岛”，争取进入全国综合治理先进城市行列，努力使青岛成为全国最安全、最稳定、最和谐的城市之一。完善经常性的严打整治机制，保持对违法犯罪分子的高压态势。加强社会稳定预警以及矛盾纠纷的排查调处、应急处置、责任追究等机制建设。坚持信访联席工作会议制度，预防和妥善处置各类群体性突发事件，完善化解人民内部矛盾的工作机制。加强基层综合治理组织建设，确保各项工作措施有效落实。加快建设公共安全应急体系，完善自然灾害、事故灾难、公共卫生、社会安全等专项应急预案，提高保障公共安全和处置突发事件的能力。强化交通、消防和安全生产管理工作，坚决遏制重特大事故的发生。开展共铸诚信活动，建立健全社会信用体系。大力整顿和规范市场经济秩序，严厉打击假冒伪劣、走私贩私、偷逃骗税、侵犯知识产权以及传销等行为。严格食品和药品安全管理，继续加大对食品药品市场的规范整顿力度，确保人民群众饮食用药安全。

　　（九）加强政府自身建设，不断提高行政能力

　　坚持科学、民主、依法决策。完善公众参与、专家论证和政府决策相结合的科学民主决策体制。建立与专家咨询委员会定期沟通制度，对涉及经济社会发展的重大问题，事先组织专家论证。继续开展“三访谈”、“市民月”活动，充分利用市民议事厅、听证会等形式，拓宽社情民意的反映渠道。对于关系人民群众切身利益的决策事项，通过向社会公布或者举行听证会等形式广泛听取意见。建立健全决策跟踪反馈制度和决策责任追究制度，实现决策权和决策责任相统一。

　　加强民主与法制建设。自觉接受人大及其常委会的监督，认真落实各项决议、决定，及时报告工作，接受质询。充分尊重和支持人民政协、各民主党派、工商联、人民团体在政治协商、民主监督和参政议政中的作用。进一步加强人大代表和政协委员提出的各项议案、建议、提案的督办落实工作，完善办理机制，提高办理质量和时效。自觉接受人民群众与新闻舆论的监督。深入开展法制教育，推进依法治市，重视司法行政建设，积极营造全社会遵法守法、依法维权的良好环境。

　　全面推进依法行政。认真贯彻落实国务院《全面推进依法行政实施纲要》，制订全面推行依法行政五年规划和年度安排，明确建设法治政府的目标和措施。进一步贯彻《行政许可法》，规范行政许可行为，加大对行政不作为、乱作为的监督力度。推行行政执法责任制，按照行政处罚与经济利益脱钩、与责任挂钩的原则，建立公开、公平、公正的评议考核制和错案责任追究制。做好第一次经济普查工作。

　　切实提高政府执行能力。大力弘扬求真务实精神，继续推进“五项工程”和“四型机关”建设，深入开展“双学三创”和“三快一提高”活动。加强各级政府领导班子建设、机关处室建设和基层建设，提高政府执行能力，提高机关处室和基层的创造力、凝聚力和战斗力，提高各级公务员为人民服务的能力。进一步优化政府工作流程，简化办事程序。抓紧建设网上审批平台，提高电子政务公共服务水平。加大效能监督力度，狠抓各项工作落实。

　　大力加强政风建设。在政府机关深入开展保持共产党员先进性教育活动，教育引导各级公务员树立正确的理想信念和高尚的道德情操，增强为民服务的意识，提高拒腐防变的能力。牢固树立以人为本、执政为民的理念，始终保持同人民群众的血肉联系。深入基层加强调查研究，体察民情，了解民意，解决实际问题。健全决策目标、执行责任、考核监督“三个体系”。进一步落实“一岗双责”责任制，严肃查处各类腐败案件，坚决纠正部门和行业不正之风。健全工程建设项目招投标、国有土地出让、产权交易、政府采购等方面的监督制度，努力从源头上预防和治理腐败。

　　各位代表！编制国民经济和社会发展第十一个五年规划，是今年的一项重要工作。我们将坚持科学化、民主化、法制化的原则，广泛征求社会各界意见建议，充分集中民智、体现民意，力求编制出一个具有高度科学性、实践性和导向性的好规划。

　　各位代表！我市经济社会发展正处在关键时期。让我们更加紧密地团结在以胡锦涛同志为总书记的党中央周围，在中共青岛市委的坚强领导下，凝聚全市人民的智慧和力量，解放思想，开拓创新，扎实苦干，为加快全市经济社会发展，完成今年各项目标任务，为提前实现全面建设小康社会的宏伟目标而努力奋斗！
